# Identifying and Evaluating Predictors of Well‐Being Among Nurses Working in Saudi Arabia: A Cross‐Sectional Study

**DOI:** 10.1155/jonm/4086275

**Published:** 2025-12-29

**Authors:** Ahmed Nahari, Monir Almotairy, Essa Hakamy, Nasreen Abdulmanna, Ghada Kurban, Turki Albraikan, Abdulmajeed Alanazi

**Affiliations:** ^1^ Medical Surgical Department, College of Nursing, King Saud University, P.O. Box 642, Riyadh, 11525, Saudi Arabia, ksu.edu.sa; ^2^ Nursing Administration and Education Department, College of Nursing, King Saud University, P.O. Box 642, Riyadh, 11525, Saudi Arabia, ksu.edu.sa; ^3^ College of Nursing, King Saud bin Abdulaziz University for Health Sciences, Jeddah, 22384, Saudi Arabia, ksau-hs.edu.sa; ^4^ King Abdullah International Medical Research Center, Jeddah, 22384, Saudi Arabia, kaimrc.med.sa; ^5^ Executive Affairs of the Ministry of Defense, Riyadh, 12478, Saudi Arabia; ^6^ College of Nursing, King Saud University, Riyadh, 11525, Saudi Arabia, ksu.edu.sa

**Keywords:** nurses, nursing staff, occupational factors, predictors, well-being, workplace

## Abstract

**Introduction:**

Nurses face numerous challenges in clinical settings that can negatively affect their well‐being. Little is known about bedside nurses’ well‐being in Saudi Arabia. Therefore, this study explored the well‐being of nurses working in Saudi Arabia and identified the contributing factors.

**Methods:**

This was a cross‐sectional study that utilized convenience sampling. Data were collected from 550 nurses working in the Saudi healthcare sector. The Nurse Well‐Being Index was utilized to measure the well‐being. Data were analyzed using descriptive statistics, analysis of variance, and logistic regression.

**Results:**

The findings showed that 74.2% of nurses were at an increased risk of poor well‐being, with frontline nurses facing a further increased risk of poor well‐being (83%). Results showed significant associations between nurses’ well‐being and level of education (*p* = 0.002), workplace (*p* < 0.001), practice setting (*p* < 0.001), working region (*p* < 0.001), bed capacity (*p* = 0.017), quality of nursing care (*p* < 0.001), and job satisfaction (*p* < 0.001). The regression analysis revealed that nurses’ well‐being was significantly influenced by years of experience (OR = 0.881, *p* = 0.027), working region–western region (OR = 2.720, *p* = 0.031), practice setting–critical care and medical–surgical (OR = 7.492, *p* < 0.001; OR = 3.222, *p* = 0.047, respectively), and quality of nursing care (OR = 4.639, *p* = 0.003).

**Discussion:**

This study revealed that most nurses were at an increased risk of poor well‐being. It also provided insights into various individual, organizational, and job‐related factors that contribute to well‐being. Future studies are needed to identify different aspects of nurses’ well‐being to develop strategies for improving their well‐being and reduce burnout and other detrimental health outcomes.

## 1. Introduction

Nurses play a vital role in strengthening healthcare systems and outcomes and they become even more prominent and necessary during emergencies and public health crises. Nurses face challenges that can affect their well‐being and ability to provide high‐quality care, particularly during emergencies and public health crises. The World Health Organization [1] has highlighted the effects of pandemics, emergencies, and disasters on raising ethical dilemmas, causing moral and psychological distress among healthcare providers that could result from the lack of resources and mental and emotional support [2–4]. As the recent coronavirus disease‐2019 (COVID‐19) pandemic unfolded, bedside nurses’ work demands increased significantly in all hospital areas, exacerbating their burdens and psychological distresses [5, 6]. This is because bedside nurses experience high workloads, frustrations, and uncertainties, which can also cause significant moral and psychological distress in their daily nursing practice and amid their efforts to provide optimal patient care [7–10]. Such negative outcomes can have devastating impacts on bedside nurses, patients, and the overall healthcare system.

According to World Health Organization [11], “Well‐being is a positive state experienced by individuals and societies. Similar to health, it is a resource for daily life and is determined by social, economic and environmental conditions. Well‐being encompasses quality of life and the ability of people and societies to contribute to the world with a sense of meaning and purpose.” Several studies have explored nurses’ well‐being during the COVID‐19 pandemic, with some showing that nurses experienced emotional exhaustion, stress, anxiety, depression, and sleep deprivation [12–15]. Others show the burdens of organizational and job‐related factors on nurses, which can influence their care outcomes and psychological health. For instance, inadequate nurse staffing has been shown to contribute to increased nurse burnout, fatigue, stress, and emotional exhaustion/burden [16–21], as well as decreased job satisfaction and performance [17]. Some specific organizational and job‐related factors, such as increased nursing workload and their practice settings, have also been associated with negative psychological and emotional effects [22–26]. Furthermore, personal characteristics (e.g., age, sex, and years of nursing experience) can also play a role in increasing psychological distress among bedside nurses [16, 23, 24, 26, 27].

In addition to the direct impact of psychological distress on nurses’ well‐being, a large and growing body of evidence indicates that poor well‐being can negatively impact patient health outcomes and healthcare utilization [28, 29]. Specifically, nurses’ poor well‐being has shown associations as follows: with poor patient safety [28]; lower patient experience quality and a negative influence on patient satisfaction, mood, and quality of life and on patient outcomes (e.g., falls, pressure ulcers, and hospital‐acquired infections [30, 31]); a negative impact on care quality, turnover rates, and costs of hiring and training new nurses, hence having significant consequences on healthcare costs and system utilization [28, 29]. Therefore, nurses’ well‐being is an integral part of the healthcare industry.

In Saudi Arabia, healthcare sector restructuring is a core element that has been gaining focus, with the final goal being to enhance the sector’s status and capabilities, thus improving the population’s general health [32]. Specifically, the strategic goals of the Saudi Health Sector Transformation Program, as part of Saudi Vision 2030, include advancing the healthcare delivery system and reducing the obstacles faced by the nursing profession [33]. However, nurses in Saudi Arabia face challenges that can have negative consequences on their health, well‐being, and the healthcare delivery system [34–37]. For instance, healthcare providers experienced significantly increased fear, anxiety, depression, and a perception of a lack of safety during the COVID‐19 pandemic [34–37]. In addition, AlAteeq et al. [38] reported that among 502 healthcare workers, depression and anxiety were highly prevalent in nurses, especially those who were female and in their 30s. The authors also emphasized the need for increased attention to mental health in nurses, as they experience depression and anxiety more often than other healthcare providers [38]. Similarly, Almaghrabi et al. [39] reported that nurses experienced increased stress, workload, and a perception of lethality compared with other healthcare providers. That is, nurses are at an increased risk for psychological health problems, which can thereby lead to negative health outcomes for both nurses and patients.

Although some studies have examined nurses’ psychological health in Saudi Arabia, the impact of individual, organizational, and job‐related factors on bedside nurses’ well‐being remains underexplored. Given the importance of related explorations, this study aimed to (1) explore the well‐being of nurses working in the Saudi healthcare sector and (2) identify contributing factors to well‐being.

### 1.1. Conceptual Framework

This study was guided by the multiperspective, ‐level, and ‐faceted model of nurses’ well‐being developed by Xiao et al. [40], which was based on an extensive systematic review and includes the following determinants: institutional determinants (e.g., healthcare reforms and institutional actors), organizational determinants (e.g., organizational practices and hospital environment), job determinants (e.g., workload), and individual determinants (e.g., demographic factors). The model posits that these are determinants of nurses’ well‐being outcomes, including happiness, relationship (e.g., work–life balance), and health (physical and mental) well‐being. In this study, we evaluated the influence of organizational determinants (e.g., work settings, bed capacity, workplace sector, and current practice settings), job determinants (e.g., staffing ratios and job position), and individual determinants (e.g., sex and years of nursing experience) on nurses’ well‐being. Furthermore, this study evaluated the influence of happiness well‐being (e.g., job satisfaction) and relationship well‐being (e.g., perceived quality of nursing care in the unit) on nurses’ well‐being. While Xiao’s model offers a comprehensive framework for understanding the interplay of personal, environmental, and contextual factors in shaping well‐being, its application can be further strengthened by integrating complementary theories. The job demands–resources (JD‐R) model provides a well‐established lens to examine how workplace demands and resources uniquely influence stress, resilience, and well‐being among nurses. Combining Xiao’s model with the JD‐R model enables a more nuanced understanding that incorporates both systemic perspectives and occupational health dynamics. This integration enhances the theoretical innovation of this study by linking individual and organizational predictors, offering a more robust explanation of nurse well‐being.

## 2. Materials and Methods

### 2.1. Study Design

This cross‐sectional study utilized convenience sampling and followed the Strengthening the Reporting of Observational Studies in Epidemiology Reporting Guidelines.

### 2.2. Study Setting and Participants

Nurses working in hospital‐ and non‐hospital‐based clinical settings in Saudi Arabia were recruited. The required sample size was estimated to be at least 500 participants to adequately power the logistic regression analysis based on the guidelines for calculating sample size for logistic regression in observational studies provided by Bujang et al. [41].

### 2.3. Inclusion and Exclusion Criteria

The inclusion criteria were as follows: (1) registered nurse, (2) currently working in the health sector in Saudi Arabia, and (3) able to read and understand English. Nursing interns and undergraduate nursing students and those who cannot speak English were excluded.

### 2.4. Data Collection

Data were collected between May 2022 and December 2023. The study questionnaire was initially distributed through an electronic survey to nursing administration departments in different health institutions and regions of Saudi Arabia. Owing to the low response rate, 750 printed questionnaires were distributed through the nursing administration departments; 493 self‐completed printed questionnaires were returned to the nursing administration departments (printed survey response rate = 65.7%). Data were collected across all work shifts to ensure representative participation. Data were double‐checked by the principal investigator to ensure accuracy. The electronic and printed surveys were managed and stored according to the requirements of the (deleted for peer review) Institutional Review Board.

### 2.5. Measures

This study administered the Nurse Well‐Being Index (NWBI), which was developed by the Mayo Clinic to measure multiple dimensions of well‐being [42]. Evidence supports its validity for assessing nurses’ well‐being [43]. To the best of our knowledge, this scale had never been used in Saudi Arabia; therefore, we invited a panel of nursing experts from academic and clinical settings to review its items for clarity and content validity. Based on their feedback, the original items of the NWBI were used without any modification.

The NWBI scale comprises nine items, with items 1–7 being yes‐or‐no items that assess fatigue, depression, burnout, anxiety, stress, mental quality of life, and physical quality of life. Item 8 assesses perceptions of the meaning of own work on a 7‐point Likert scale, where 1 is “Very Strongly Disagree” and 7 is “Very Strongly Agree.” Item 9 evaluates the work schedule and family/personal life balance on a 5‐point Likert scale, where 1 is “Strongly Disagree” and 5 is “Strongly Agree.” The total score for the NWBI ranges from −2 (lowest risk) to 9 (highest risk), with higher scores indicating a higher risk of poor well‐being [42]. Based on Dyrbye et al. [43], the NWBI results were categorized into high (scores ≥ 2) and low risks (scores between ≥ −2 and > 2) of poor well‐being, and the reliability of the overall scale was acceptable in this study (Cronbach’s alpha = 0.74).

The questionnaire also included items on demographic characteristics such as sex, education level, workplace sector (Ministry of Health vs. non‐Ministry of Health), current practice setting, current position (frontline nursing staff vs. other nonfrontline nursing staff), bed capacity, nurse‐to‐patient ratio in the last shift, average nurse‐to‐patient ratio in the unit in the last working week, and region of the workplace.

Two NWBI items that evaluate perceptions of the quality of nursing care in their unit and their overall job satisfaction were also used. The quality of nursing care in the unit item was rated on a 4‐point Likert scale, where 1 was “Poor,” 2 was “Fair,” 3 was “Good,” and 5 was “Excellent.” The overall job satisfaction item was scored on a 4‐point Likert scale, where 1 was “Very Dissatisfied,” 2 was “Moderately Dissatisfied,” 3 was “Moderately Satisfied,” and 4 was “Very Satisfied.”

### 2.6. Data Analysis

Descriptive statistics were used to report the means and standard deviations for continuous measures and frequencies for categorical measures. Inferential statistics (Pearson’s chi‐square and Cramer’s V correlation tests) were used to determine the significance of differences in NWBI categories by the quality of nursing care in their unit, job satisfaction, and demographic characteristics.

A multivariable binary logistic regression analysis was performed. Two regression models were developed to explore the impact of the variables of interest on nurses’ well‐being. The first model included all participants and explored the impact of the variables of interest on the well‐being of all nurses regardless of position or practice setting. The second model included only those participants who reported practicing as frontline nurses in hospital settings. In each model, model diagnostics were conducted to assess the fit of the regression models, specifically the Hosmer–Lemeshow goodness‐of‐fit test, the Nagelkerke *R* ^2^, and classification accuracy. Variables were selected for inclusion in the regression models based on theoretical relevance and statistical significance (*p* < 0.10) in the bivariate analyses. Statistical significance was set at a *p* < 0.05, and all statistical analyses were performed using SPSS for Windows, version 29.0. Finally, several variables, including sex and education, had approximately 10% missing data. No imputation was performed, as the proportion of missing data was below the commonly accepted threshold (i.e., < 20%) for which imputation is typically recommended. Instead, analyses were conducted using available cases (listwise deletion).

### 2.7. Ethical Considerations

This study was approved by the Institutional Review Board of King Saud University (IRB# E‐21‐6023). The study was conducted in accordance with the principles outlined in the Declaration of Helsinki. Prior to study enrollment, an electronic informed consent was obtained from all participants who completed the electronic survey, while those who completed the printed survey provided written informed consent. This research was conducted according to the principles outlined in the Declaration of Helsinki and its later amendments. Participants were informed that their participation was voluntary and they had the right to withdraw from the study at any time without any consequences. No identifiable information was obtained from participants.

## 3. Results

The analysis included a total of 550 nurses. As shown in Table 1, the participants were relatively young with a mean age of 33 ± 6.03 years. Among the participants, 69.5% were female; 64.4% had a bachelor’s degree as their highest education level; 76.7% were working for health facilities under the Ministry of Health; 74.9% were working in hospital settings; 26.7% and 24.5% were working in the emergency department and critical care settings, respectively; 52.2% were working in facilities with ≤ 200 beds; 61.8% were frontline nursing staff; 41.1% reported a nurse‐to‐patient ratio in their last shift that met the Saudi Patient Safety Center’s (SPSC) staffing ratio requirements and 43.3% reported an average ratio that did not meet the SPSC’s requirements; 50.5% rated the quality of nursing care in their unit as good; 62.9% were satisfied with their job; 74.2% were classified as being at a high risk of poor well‐being; and 41.5% were from the western region of Saudi Arabia.

**Table 1:** Sample characteristics.

Variables	Overall sample (*n* = 550) *n* (%) or mean ± SD	Frontline nurses (*n* = 264) *n* (%) or mean ± SD
Age (in years)	32.95 ± (6.03)	31.84 ± (5.26)

*Sex*		
Male	116 (21.1%)	43 (16.3%)
Female	382 (69.5%)	213 (80.7%)
Missing	52 (9.5%)	8 (3.0%)

*Education level*		
Diploma/associate degrees	91 (16.5%)	28 (10.6%)
Bachelor’s degree	354 (64.4%)	212 (80.3%)
Postgraduate degrees	53 (9.6%)	23 (8.7%)
Missing	52 (9.5%)	1 (0.4%)

*Workplace sector*		
Ministry of Health	422 (76.7%)	234 (88.6%)
Non‐Ministry of Health	72 (13.0%)	29 (11.0%)
Missing	56 (10.1%)	1 (0.4%)

*Current workplace setting*		
Nonhospital	82 (14.9%)	0 (0%)
Hospital	412 (74.9%)	264 (100%)
Missing	56 (10.2%)	0 (0%)

*Practice setting*		
Outpatient	84 (15.4%)	0 (0%)
Critical care	135 (24.5%)	104 (39.4%)
Emergency settings	147 (26.7%)	90 (34.1%)
Medical–surgical	94 (17.1%)	70 (26.5)
Other hospital settings	34 (6.2%)	0 (0%)
Missing	56 (10.2%)	0 (0%)

*Bed capacity*		
≤ 200 beds	287 (52.2%)	125 (47.3%)
201–500 beds	159 (29.0%)	113 (42.8%)
> 500 beds	35 (6.4%)	17 (6.4%)
Missing	69 (12.5%)	9 (3.4%)

*Nurse-to-patient ratio in last shift*		
Yes	127 (23.1%)	93 (35.2%)
No	226 (41.1%)	162 (61.4%)
Missing	97 (35.8%)	9 (3.4%)

*Nurse-to-patient ratio in unit*		
Yes	119 (21.6%)	89 (33.7%)
No	238 (43.3%)	163 (61.7%)
Missing	193 (35.1%)	12 (4.5%)

*Quality of nursing care*		
Poor	28 (5.1%)	13 (4.9%)
Fair	93 (16.9%)	54 (20.45%)
Good	278 (50.5%)	152 (57.6%)
Excellent	101 (18.4%)	45 (17.0%)
Missing	50 (9.1%)	0 (0%)

*Job satisfaction*		
Satisfied	346 (62.9%)	177 (67.0%)
Dissatisfied	155 (28.1%)	87 (33.0%)
Missing	49 (8.9%)	0 (0%)

*Region*		
Eastern	39 (7.1%)	25 (9.5%)
Western	228 (41.5%)	93 (35.2%)
Central	101 (18.4%)	60 (22.7%)
South	70 (12.7%)	48 (18.2%)
Missing	112 (20.4%)	3 (1.1%)

*Well-being*		
Low risk (−2 to 1)	115 (20.9%)	45 (17.0%)
High risk (2 to 9)	408 (74.2%)	219 (83.0%)
Missing	27 (4.9%)	0 (0%)

Among frontline nurses (*n* = 264), the mean age was 31.8 ± 5.26 years, 80.7% were female; 80.3% had a bachelor’s degree as their highest education level; 88.6% were working for health facilities under the Ministry of Health; 34.1% and 39.4% were working in emergency department and critical care settings, respectively; 47.3% were working in facilities with ≤ 200 beds; 61.4% reported a nurse‐to‐patient ratio in the last shift that met the SPSC requirements, and 61.7% reported that the average nurse‐to‐patient ratio in their units did not meet the SPSC requirements; 57.6% rated the quality of nursing care in their unit as good; 67.0% were satisfied with their job; 83.0% were classified as being at a high risk of poor well‐being; and 35.2% were from the western region of Saudi Arabia.

In the overall study sample (Table 2), Pearson’s chi square or Cramer’s V correlation tests showed statistically significant associations of NWBI risk status with the following factors: education level (Cramer’s V = 0.158, *p* = 0.002), hospital and nonhospital workplace settings (*χ* ^2^ = 18.78, *p* < 0.001), practice setting (Cramer’s V = 0.254, *p* < 0.001), region of workplace (Cramer’s V = 0.200, *p* < 0.001), bed capacity (Cramer’s V = 0.131, *p* = 0.017), quality of nursing care in their unit (Cramer’s V = 0.200, *p* < 0.001), and job satisfaction (*χ* ^2^ = 23.09, *p* < 0.001).

**Table 2:** Relationships between nurses’ well‐being and other factors among the overall sample (*N* = 550).

		Risk category		Chi‐square or Cramer’s V	
		Low risk *n* (%)	High risk *n* (%)	Test value	*p* value
Sex	Male	27 (24.1%)	89 (23.1%)	0.048	0.827
	Female	85 (75.9%)	296 (76.9%)		

Education level	Diploma/Associate’s degree	31 (27.7%)	60 (15.6%)	0.158	0.002^∗∗^
	Bachelor’s degree	76 (67.9%)	277 (71.9%)		
	Master’s degree or Higher	5 (4.5%)	48 (12.5%)		

Working sector	Ministry of Health	99 (88.4%)	322 (83.9%)	1.39	0.238
	Non‐Ministry of Health	13 (11.6%)	62 (16.1%)		

Workplace	Hospital	76 (69.7%)	335 (87.2%)	18.78	< 0.001^∗∗^
	Nonhospital	33 (30.3%)	49 (12.8%)		

Current practice setting	Outpatient	35 (32.1%)	49 (12.8%)	0.254	< 0.001^∗∗^
	Inpatient critical care	16 (14.7%)	118 (30.7%)		
	Emergency settings	26 (23.9%)	121 (31.5%)		
	Medical–surgical	20 (18.3%)	74 (19.3%)		
	Other hospital settings	12 (11.0%)	22 (5.7%)		

Region of workplace	Central	14 (13.9%)	87 (25.9%)	0.200	< 0.001^∗∗^
	Western	63 (62.4%)	164 (48.8%)		
	Eastern	2 (2.0%)	37 (11.0%)		
	Southern	22 (21.8%)	48 (14.3%)		

Bed capacity	≤ 200 beds	73 (68.9%)	213 (57.0%)	0.131	0.017^∗^
	201–500 beds	23 (21.7%)	136 (36.4%)		
	> 501 Beds	10 (9.4%)	25 (6.7%)		

Quality of nursing care	Poor	4 (3.6%)	24 (6.2%)	0.200	< 0.001^∗∗^
	Fair	10 (8.9%)	82 (21.2%)		
	Good	61 (54.5%)	217 (56.1%)		
	Excellent	37 (33.0%)	64 (16.5%)		

Job satisfaction	Satisfied	98 (87.5%)	247 (63.7%)	23.09	< 0.001^∗∗^
	Dissatisfied	14 (12.5%)	141 (36.3%)		

^∗^ *p* ≤ 0.05.

^∗∗^ *p* ≤ 0.01.

Among frontline nurses (Table 3), the correlation tests showed statistically significant associations for NWBI risk status with the quality of nursing care in their unit (Cramer’s V = 0.266, *p* < 0.001) and job satisfaction (*χ* ^2^ = 14.22, *p* < 0.001).

**Table 3:** Relationships between nurses’ well‐being and other factors among frontline nurses (*n* = 264).

		Risk category		Chi‐square or Cramer’s V	
		Low risk *n* (%)	High risk *n* (%)	Test value	*p* value
Sex	Male	4 (8.9%)	3 (17.8%)	2.18	0.14
	Female	41 (91.1%)	180 (82.2%)		

Education level	Diploma/associate degree	6 (13.3%)	22 (10.1%)	0.109	0.211
	Bachelor’s degree	38 (84.4%)	174 (79.8%)		
	Master’s degree or higher	1 (2.2%)	22 (10.1%)		

Working sector	Ministry of Health	43 (95.6%)	191 (87.6%)	2.40	0.122
	Non‐Ministry of Health	2 (4.4%)	27 (12.4%)		

Practice setting	Critical care	12 (26.7%)	92 (42.0%)	0.119	0.154
	Emergency settings	18 (40.0%)	72 (32.9%)		
	Medical–surgical	15 (33.3%)	55 (25.1%)		

Region of workplace	Central	10 (25.6%)	50 (49.6%)	0.130	0.283
	Western	18 (46.2%)	75 (40.1%)		
	Eastern	1 (2.6%)	24 (12.8%)		
	Southern	10 (25.6%)	38 (20.3%)		

Bed capacity	≤ 200 beds	25 (58.1%)	100 (47.2%)	0.141	0.078
	201–500 beds	13 (30.2%)	100 (47.2%)		
	> 501 Beds	5 (11.6%)	12 (5.7%)		

Nurse‐to‐patient ratio in the last shift	Yes	13 (30.2%)	80 (37.7%)	0.87	0.351
	No	30 (69.8%)	132 (37.7%)		

Nurse‐to‐patient ratio in unit	Yes	15 (34.1%)	74 (35.6%)	0.035	0.851
	No	29 (65.9%)	134 (64.4%)		

Quality of nursing care	Poor	0 (0%)	13 (5.9%)	0.266	< 0.001^∗∗^
	Fair	3 (6.7%)	51 (23.3%)		
	Good	26 (57.8%)	126 (57.5%)		
	Excellent	16 (35.6%)	29 (13.2%)		

Job satisfaction	Satisfied	41 (91.1%)	136 (62.1%)	14.22	< 0.001^∗∗^
	Dissatisfied	4 (8.9%)	83 (37.9%)		

^∗^ *p* ≤ 0.05.

^∗∗^ *p* ≤ 0.01.

The findings of the regression analysis of the first model indicated that years of nursing experience, region of workplace, practice setting, and quality of nursing care in their unit significantly impacted nurses’ well‐being (Table 4). After adjusting for other variables in the model, the odds of being at a high risk for poor well‐being decreased by 11.9% (odds ratio [OR] = 0.881, 95% confidence interval [CI] = 0.787, 0.985, *p* = 0.027), with each unit increase in years of nursing experience. The odds of having a high risk for poor well‐being were 172% higher (OR = 2.720, 95% CI [1.097, 6.745], *p* = 0.031) for nurses practicing in the western region than for those practicing in the southern region after adjusting for other variables in the model. Nurses in critical care and medical–surgical care settings had 649% and 222% higher odds, respectively, of being at a high risk of poor well‐being (OR = 7.492, 95% CI [2.216, 25.331], *p* < 0.001; and OR = 3.222, 95% CI = [1017, 10.210], *p* = 0.047, respectively) than those practicing in outpatient care settings—after adjusting for other variables in the model. Nurses who rated the quality of nursing care delivered in their unit as poor or fair had 363% higher odds of being at a high risk of poor well‐being (OR = 4.639, 95% CI [1.696, 12.691], *p* = 0.003) than those who rated it as good or excellent (after adjusting for other variables in the model).

**Table 4:** Multivariable logistic regression models for nurses’ well‐being.

Variables	Model 1 (*n* = 310)	Model 2 (*n* = 202)
	Odds ratio (95% CI)	Odds ratio (95% CI)
Age (in years)	1.028 (0.938, 1.126)	0.990 (0.867, 1.130)
Sex^a^	0.971 (0.416, 2.264)	2.984 (0.646, 13.778)
Education level^b^	0.692 (0.285, 1.680)	0.669 (0.165, 2.719)
Years of experience	0.881 (0.787, 0.985)^∗^	0.927 (0.786, 1.092)
Years of Experience in the current position	1.052 (0.969, 1.141)	1.116 (0.987, 1.262)
Practice sector^c^	1.813 (0.580, 5.663)	1.337 (0.241, 7.854)
Region of workplace		
Central^d^	2.080 (0.660, 6.551)	1.693 (0.515, 5.562)
Western^e^	2.720 (1.097, 6.745)^∗∗^	1.273 (0.343, 4.773)
Eastern^f^	5.271 (0.870, 31.943)	3.546 (0.334, 37.643)
Practice setting		
Critical care^g^	7.492 (2.216, 25.331)^∗^	—
Emergency care^h^	2.653 (0.790, 8.912)	—
Medical–surgical care^i^	3.222 (1.017, 10.210)^∗^	—
Other hospital care^j^	1.387 (0.367, 5.247)	—
Bed capacity		
200 beds^k^	0.901 (0.228, 3.567)	1.724 (0.293, 10.166)
201–500 beds^l^	1.542 (0.378, 6.284)	3.581 (0.665, 19.295)
Being frontline nurse^m^	0.864 (0.358, 2.081)	—
Job satisfaction^n^	0.495 (0.220, 1.114)	0.265 (0.080, 0.872)^∗^
Quality of nursing care in unit^o^	4.639 (1.696, 12.691)^∗∗^	7.168 (1.480, 34.705)^∗^
Nurse‐to‐patient ratio in last shift^p^	—	0.568 (0.159, 2.033)
Nurse‐to‐patient ratio in the unit^q^	—	0.953 (0.253, 3.589)

^a^Sex: female vs. male.

^b^Education level: bachelor’s degree or higher degree vs. diploma or associate degree.

^c^Practice sector: Ministry of Health facilities vs. Non‐Ministry of Health facilities.

^d^Western region: practicing in the western region vs. practicing in the central region.

^e^Eastern region: practicing in the eastern region vs. practicing in the central region.

^f^Southern region: practicing in the southern region vs. practicing in the central region.

^g^Critical care settings: critical care vs. outpatient care.

^h^Emergency care settings: emergency care vs. outpatient care.

^i^Medical–surgical care settings: medical–surgical care vs. outpatient care.

^j^Other hospital care settings: other hospital care settings vs. outpatient care settings.

^k^Capacity of 201–500 beds: bed capacity of 201–500 beds vs. bed capacity of < 201 beds.

^l^Capacity of > 500 beds: bed capacity of > 500 beds vs. bed capacity of < 201.

^m^Being frontline nurse: frontline nurse vs. nonfrontline nurse.

^n^Job satisfaction: satisfied vs. dissatisfied.

^o^Quality of nursing care in unit: good or excellent vs. poor or fair.

^p^Nurse‐to‐patient ratio in the last shift: meeting vs. not meeting the requirements.

^q^Nurse‐to‐patient ratio in unit: meeting vs. not meeting the requirements.

^∗^ *p* ≤ 0.05.

^∗∗^ *p* ≤ 0.01.

In the second regression model, including only frontline nurses, the quality of nursing care in the unit remained significant and the impact of job satisfaction became significant (Table 4). That is, nurses who rated the quality of nursing care in their unit as poor or fair had 616% higher odds of being at a high risk for poor well‐being (OR = 7.168, 95% CI [1.480, 34.705], *p* = 0.014) than those who rated the nursing care as good or excellent, after adjusting for other variables in the model. Nurses satisfied with their current job had 73.5% lower odds of being at a high risk for poor well‐being (OR = 0.265, 95% CI [0.080, 0.872], *p* = 0.029) compared to those who were not satisfied, after adjusting for other variables in the model. The nurse‐to‐patient ratio according to SPSC requirements and other variables showed no significant findings in the regression model.

## 4. Discussion

Nurses working in clinical settings experience various challenges that can negatively affect their well‐being and ability to provide high‐quality care. This study examined the well‐being and its contributing factors among nurses working in Saudi Arabia’s healthcare sector. The results indicated that most nurses were at an increased risk of poor well‐being. These findings are consistent with those of previous studies that examined the psychological health and well‐being of healthcare providers, including nurses, working in Saudi Arabia [34–38, 40]. There is evidence from other countries that also depicts nurses experiencing numerous psychological distresses such as burnout, fatigue, and low mental/physical quality of life [5, 44, 45]. It should be noted here that previous research revealed that a large percentage of nurses reported high levels of burnout, dissatisfaction, and intent to leave their employers before and during the COVID‐19 pandemic, with these outcomes being worse (or remaining high) during the pandemic [44].

Moreover, according to our findings that the standard nurse‐to‐patient ratios recommended by the SPSC in collaboration with the International Council of Nurses and the Saudi Ministry of Health were most often not met (including among frontline nurses working in critical care and emergency departments), it may be that the increased risk of poor well‐being observed in our sample may be due to staffing shortages. Indeed, a bulk of studies have underpinned staff shortages and high workloads as major contributing factors to burnout, high turnover rates, and psychological distress [46]. In the present study, more than half of the nurses worked in critical care units and emergency departments; meanwhile, in a study by Aiken et al. [44], higher rates of burnout, dissatisfaction, and intent to leave were observed among nurses working in critical care units and emergency departments, especially during the COVID‐19 pandemic. Thus, the noncompliance of nurse‐to‐patient ratios with the SPSC requirements and some practice settings may have contributed to the increased risk of poor well‐being observed in our study.

Our study findings provide insights into the individual, organizational, and job‐related factors impacting the well‐being of nurses working in Saudi Arabia’s healthcare sector, which are aligned with the study framework. Among individual factors, years of nursing experience impacted nurses’ well‐being, similarly to the findings in the study by Al Sabei et al. [47], wherein there was a lower well‐being among nurses with more years of experience. Educational level also impacted well‐being in our sample, which is consistent with the evidence in Al‐Hanawi et al.’s [35] study in Saudi Arabia. However, while Al‐Hanawi et al.’s [35] findings demonstrated higher risks of psychological distress among female and younger nurses, no significant impact of sex and age on well‐being was observed in the current study’s sample.

Aligned with the study framework, our results showed that organizational and job‐related factors such as the workplace, practice setting, bed capacity, and quality of nursing care in their unit played significant roles in nurses’ well‐being. These findings mirror those of previous studies, where there was an impact of organizational and job‐related factors on nurses’ well‐being [44, 48–50]. However, our study showed that nurse‐to‐patient ratios among frontline nurses practicing in hospital settings did not significantly impact their well‐being. It should be noted here that most nurses in our sample reported that their units did not meet the SPSC nurse‐to‐patient standard, which might have obscured the potential relationship between nurse‐to‐patient ratios and nurses′ wellbeing, making it more difficult to detect any significant effects.

Our results showed a high risk of poor well‐being among nurses working in Saudi Arabia’s healthcare sector and highlighted several important factors that could increase this risk. These findings raise concerns about not only the psychological health and well‐being of nurses but also the negative health outcomes associated with these working conditions at the patient and healthcare delivery system levels. Previous studies examining the links between nurses’ well‐being and patient outcomes revealed negative patient outcome consequences associated with poor nurses’ well‐being [30, 51]. Psychological distress and stressors faced by nurses in healthcare settings have also been associated with adverse patient outcomes including patient falls, nosocomial infections, and medication errors [30, 51]. Moreover, nurses’ poor well‐being is associated with increased healthcare expenditures and suboptimal healthcare delivery and utilization [29].

### 4.1. Implications for Practice and Future Research

The findings of this study support the importance of developing strategies to improve nursing work environments and minimize burnout and other detrimental health outcomes among nurses. These strategies stand to benefit patients’ health outcomes and may help secure an optimized healthcare delivery process, improved nursing work environment, and the contribution to national workforce planning and nurse retention efforts, both of which are central to Saudi Arabia’s Vision 2030 objectives for healthcare transformation.

Enhanced work environments, equitable staffing, and supportive leadership can help retain skilled nurses and foster higher levels of engagement and productivity. These improvements not only promote nurses’ physical and psychological well‐being but also contribute to better patient outcomes, continuity of care, and greater system efficiency—key priorities within Vision 2030s focus on quality and sustainability in healthcare delivery.

Our findings also highlighted the need for more research on the potential contributing factors to nurses’ well‐being. Further progress is greatly required in determining the potential individual, organizational, and job‐related factors influencing nurses’ well‐being. Another possible area of scrutiny for future research is the impact of nurses’ well‐being on patient health outcomes and the healthcare delivery system in Saudi Arabia.

### 4.2. Limitations

This study has various limitations. First, the study design, which may have introduced social desirability bias (e.g., through favorable/unfavorable rating of nurses’ well‐being status and nurse‐to‐patient ratios). Future studies should utilize alternative methodologies that mitigate the impact of social desirability bias. Second, the study utilized a convenience sampling approach, which may limit the generalizability of our findings. The data collected in this study were based on self‐reports, which may be subject to social desirability bias. This sampling method may potentially affect the accuracy of the results. Future research should employ more rigorous sampling techniques to obtain more representative samples. Finally, we limited this research to measuring certain aspects of well‐being. Therefore, future qualitative studies are needed to assess other aspects of nurses’ mental health, contributing factors to well‐being that are relevant to the Saudi social and healthcare contexts, and potential subsequent effects on healthcare outcomes.

## 5. Conclusions

Nurses’ well‐being is fundamental to sustaining safe, effective, and high‐quality healthcare services, particularly during times of crisis such as pandemics. This study highlights that nurses face a considerable risk of poor well‐being due to a combination of individual, organizational, and job‐related factors, including workload, support systems, and work environment conditions. These findings emphasize the urgent need for healthcare institutions and policymakers to prioritize nurses’ well‐being as a critical component of workforce stability and patient safety.

Investing in supportive work environments, fair staffing policies, and professional development opportunities can enhance both nurse satisfaction and retention, contributing to improved healthcare system performance. Moreover, the insights gained from this study can inform evidence‐based interventions and strategic policies that align with Saudi Arabia’s Vision 2030, particularly its goals of advancing healthcare quality, efficiency, and workforce empowerment.

Future research should build upon these findings by exploring the mechanisms linking nurses’ well‐being to patient outcomes, as well as evaluating targeted interventions designed to strengthen resilience, reduce burnout, and promote sustainable workforce practices. A comprehensive understanding of these relationships will be vital to ensuring that nurses remain healthy, motivated, and capable of delivering optimal care to the populations they serve.

## Ethics Statement

This study was approved by the Institutional Review Board of King Saud University (IRB# E‐21‐6023). Informed consent was obtained from all participants prior to data collection. This study adhered to the principles of the Declaration of Helsinki.

## Disclosure

All authors have read and agreed to the submitted version of the manuscript.

## Conflicts of Interest

The authors declare no conflicts of interest.

## Author Contributions

Conceptualization, Ahmed Nahari, Monir Almotairy, Ghada Kurban, Nasreen Abdulmanna, and Turki Albraikan; methodology, Ahmed Nahari, Monir Almotairy, Ghada Kurban, Nasreen Abdulmanna, and Turki Albraikan; software, Ahmed Nahari and Monir Almotairy; validation, Ahmed Nahari and Monir Almotairy; formal analysis, Ahmed Nahari and Monir Almotairy; investigation, Ahmed Nahari and Monir Almotairy; data curation, Monir Almotairy and Abdulmajeed Alanazi; writing–original draft preparation and review and editing, Ahmed Nahari, Monir Almotairy, Ghada Kurban, Nasreen Abdulmanna, Turki Albraikan, Abdulmajeed Alanazi, and Essa Hakamy.

Ahmed Nahari and Monir Almotairy contributed equally as first joint authors.

## Funding

This study was funded by the Ongoing Research Funding Program (Project No. ORF‐2025‐1133) and King Saud University, Riyadh, Saudi Arabia.

## Data Availability

The data presented in this study are available on request from the corresponding author. The data are not publicly available due to privacy or ethical restrictions.
